# Proteomic Profiling Reveals the Architecture of Granulomatous Lesions Caused by Tuberculosis and *Mycobacterium avium* Complex Lung Disease

**DOI:** 10.3389/fmicb.2019.03081

**Published:** 2020-01-17

**Authors:** Shintaro Seto, Kozo Morimoto, Tsutomu Yoshida, Miyako Hiramatsu, Minako Hijikata, Toshi Nagata, Fumihito Kikuchi, Yuji Shiraishi, Atsuyuki Kurashima, Naoto Keicho

**Affiliations:** ^1^Department of Pathophysiology and Host Defense, Research Institute of Tuberculosis, Japan Anti-Tuberculosis Association, Tokyo, Japan; ^2^Respiratory Disease Center, Fukujuji Hospital, Japan Anti-Tuberculosis Association, Tokyo, Japan; ^3^Department of Health Science, Hamamatsu University School of Medicine, Hamamatsu, Japan; ^4^Department of Pathology, Fukujuji Hospital, Japan Anti-Tuberculosis Association, Tokyo, Japan; ^5^Research Institute of Tuberculosis, Japan Anti-Tuberculosis Association, Tokyo, Japan

**Keywords:** tuberculosis, *Mycobacterium avium* complex lung disease, granuloma, necrotic caseum, proteomics

## Abstract

Tuberculosis (TB) and *Mycobacterium avium* complex lung disease (MAC-LD) are both characterized pathologically by granuloma lesions, which are typically composed of a necrotic caseum at the center surrounded by fibrotic cells and lymphocytes. Although the histological characterization of TB and MAC-LD granulomas has been well-documented, their molecular signatures have not been fully evaluated. In this research we applied mass spectrometry-based proteomics combined with laser microdissection to investigate the unique protein markers in human mycobacterial granulomatous lesions. Comparing the protein abundance between caseous and cellular sub-compartments of mycobacterial granulomas, we found distinct differences. Proteins involved in cellular metabolism in transcription and translation were abundant in cellular regions, while in caseous regions proteins related to antimicrobial response accumulated. To investigate the determinants of their heterogeneity, we compared the protein abundance in caseous regions between TB and MAC-LD granulomas. We found that several proteins were significantly abundant in the MAC-LD caseum of which proteomic profiles were different from those of the TB caseum. Immunohistochemistry demonstrated that one of these proteins, Angiogenin, specifically localized to the caseous regions of selected MAC-LD granulomas. We also detected peptides derived from mycobacterial proteins in the granulomas of both diseases. This study provides new insights into the architecture of granulomatous lesions in TB and MAC-LD.

## Introduction

Tuberculosis (TB) is a major infectious disease worldwide, causing high mortality and morbidity. There were 10 million new cases worldwide in 2017, with 1.6 million deaths, including 0.3 million among people with human immunodeficiency virus (WHO, 2018). TB is characterized pathologically by lung granulomatous inflammation. The causative pathogen, *Mycobacterium tuberculosis*, is inhaled into the respiratory tract, and then phagocytosed by alveolar macrophages. Once internalized into the macrophages, *M. tuberculosis* bacilli actively inhibit phagolysosome biogenesis, ensuring their survival. Infected macrophages trigger inflammatory responses to induce the recruitment of uninfected macrophages and the formation of cell aggregations, which are known as granuloma. Upon the development of adaptive immunity, lymphocytes surround the macrophage-rich granulomatous lesions to contain the infected bacilli. T lymphocytes around granulomas secrete inflammatory cytokines including IFN-γ, which activate *M. tuberculosis*-infected macrophages and facilitate the eradication of infected bacilli in granulomas. In some granulomas, however, *M. tuberculosis*-infected macrophages fall into necrosis, followed by the formation of necrotic caseum at the center (Russell, 2001; Ramakrishnan, 2012).

Non-tuberculous mycobacteria (NTM) cause chronic pulmonary or extrapulmonary granulomatous diseases. Of the NTM-related diseases, *Mycobacterium avium* complex lung disease (MAC-LD) is the most common, with its incidence and prevalence increasing globally (Adjemian et al., 2012; Hoefsloot et al., 2013; Namkoong et al., 2016). Two radiological types of MAC-LD—fibrocavitary (FC) and nodular bronchiectatic (NB)—have been reported, with quite different clinical features. Nevertheless, the hallmark of MAC-LD is the presence of extensive granulomatous lesions (Fujita et al., 1999; Griffith et al., 2007).

The pathophysiology of necrotic caseum in TB granulomas has been investigated. The necrotic caseum is hypoxic and rich in bactericidal activity, and thus is thought to effectively eliminate infected mycobacterial bacilli. However, this region also promotes inflammation and tissue damage, and undergoes liquefaction resulting in cavitation, which facilitates bacterial dissemination and the transmission of the disease (Russell, 2007; Ramakrishnan, 2012). The hypoxic environment induces changes of gene expression in *M. tuberculosis*, leading to the acquisition of a persistent or dormant phenotype (Voskuil et al., 2003). The rate of penetration into the necrotic caseum differs among anti-TB drugs, leading to incomplete sterilization of *M. tuberculosis* bacilli (Dartois, 2014). These observations suggest that the necrotic caseum plays a critical role in the development, maintenance, and exacerbation of TB. The histological appearance of MAC-LD granulomas has been reported to be indistinguishable from that of TB (Snijder, 1965; Marchevsky et al., 1982), but the pathophysiology of the necrotic caseum of MAC-LD granulomas has not yet been assessed in detail.

To elucidate the molecular structure of TB and MAC-LD granulomatous lesions, we employed formalin-fixed paraffin-embedded (FFPE) specimens of surgically resected lung tissues, and obtained information about their component proteins by a combination of laser microdissection (LMD) and mass spectrometry-based proteomics. We analyzed the protein abundance in sub-compartments of granulomas, and found heterogeneity of the caseum between TB and MAC-LD. We also detected peptides derived from mycobacterial bacilli in granulomas. These results demonstrated that proteomic analysis in combination with LMD is an effective approach to the molecular dissection of mycobacterial granulomatous lesions.

## Materials and Methods

### Ethics Statement

All study participants gave written informed consent, and this study was approved by Fukujuji Hospital Institutional Review Board (IRB) and the Research Institute of Tuberculosis IRB. In the surgical treatment of TB and multidrug-resistant TB, patients were carefully selected and managed according to the criteria and guidelines (Shiraishi et al., 2004; WHO, 2008). For MAC-LD, the indications for surgery include a poor response to drug therapy, the development of macrolide-resistant disease, or the presence of a significant disease-related complication such as hemoptysis (Griffith et al., 2007).

### Sample Preparation

Following pathologic inspection of surgically resected lung tissues, 10-μm thick sections from FFPE samples containing granulomatous lesions were sliced with a microtome and mounted onto PEN membrane slides (Zeiss). Sections were deparaffinized and stained with hematoxylin, and granulomatous lesions were dissected under an LMD7000 (Leica). Collected tissue samples were lysed with Liquid Tissue^TM^ MS Protein Prep kits (Expression Pathology) in accordance with the manufacturer’s instructions. The dissected FFPE samples, suspended in the lysis solvent, were incubated at 95°C for 90 min, and then cooled on ice for 3 min. Trypsin (1/50 w/w) was added followed by incubation at 37°C overnight. Dithiothreitol was added to a final concentration of 10 mM, and the samples were heated for 5 min at 95°C. Samples were then desalted using MonoSpin^TM^ C18 column (GL Science) and dried, followed by redissolution with 0.1% formic acid and filtration with Ultrafree-MC column (5 μm, Merck Millipore).

### Liquid Chromatography Coupled to Tandem Mass Spectrometry (LC–MS/MS)

The samples containing 3 μg of peptides were analyzed by LC–MS/MS using an EASY-nLC 1000 (Thermo Fisher Scientific) and a Q Exactive mass spectrometer (Thermo Fisher Scientific). For full capture of MS and MS/MS events, resolutions of 70,000 and 17,500 were used, respectively. Full-scan MS data were acquired using a mass range of 350–1800 m/z. Peptide separation was carried out using an analytical column NTCC-360/75-3-125 (C18, particle diameter 3 μm, 0.075 mm × 125 mm, Nikkyo Technos) with reverse-phase LC elution. The mobile phases were 0.1% formic acid (A), and 0.1% formic acid in acetonitrile (B). Peptides were eluted at a flow rate of 300 nl/min with an increasing linear gradient from 0 to 35% of B for 240 min.

### Bioinformatics

Mass spectrometry raw files were processed with MaxQuant (version 1.6.0.16) software and searched with the built-in Andromeda search engine (Cox and Mann, 2008) against UniProt sequences UP000005640 for identification of human proteins; UP000001584 for *M. tuberculosis* proteins; and UP000218842, UP000223089, and UP000225854 for *M. avium* subsp. *hominissuis* proteins. The parameters for the search were set to Trypsin/P enzyme with up to 2 missed cleavages allowed; N-acetylation of protein and oxidation of methionine were set to variable modification; a false discovery rate (FDR) of less than 0.01 for protein and peptide was allowed; and the required minimum peptide length was seven amino acids. For quantitative proteomics, we used the label-free quantification (LFQ) algorithm (Cox et al., 2014) included in the MaxQuant software using the following parameters: match between runs was set to 0.7 min window; LFQ minimum-ratio count was set to 1.

Datasets (Supplementary Table S1) were analyzed by Perseus software (version 1.6.0.7) (Tyanova et al., 2016). LFQ intensity values were log_2_-transformed, and missing values were replaced by random numbers drawn from a normal distribution (downshift 1.8, width 0.3). Principal component analysis (PCA) and hierarchical clustering were performed on these datasets. Differentially abundant proteins were identified using the cutoff of Welch’s *t*-test with the FDR at 0.05 and an absolute value of fold change of 5. For gene ontology (GO) term enrichment analysis, selected datasets were uploaded to DAVID (Huang da et al., 2009) and Metascape (Zhou et al., 2019). Venn diagram analysis, hierarchical clustering analysis, the *post hoc* Tukey–Kramer test following the one-way analysis of variance test (ANOVA) and Student’s *t*-test were performed using R ver. 3.5.1.

### Data Availability

Mass spectrometry raw files have been deposited to the ProteomeXchange consortium via the jPOST (Okuda et al., 2017) partner repository with the dataset identifier PXD014086/JPST000609.

### Immunohistochemistry

For immunohistochemistry, 4-μm thick sections of FFPE tissues on glass slides were deparaffinized, then dehydrated and rehydrated. The slides were subjected to antigen retrieval using an autoclave at 105°C for 1 min in 10 mM sodium citrate (pH 6.0) and 0.05% Tween 20. Slides were washed twice with 0.025% Triton-X100 in TBS for 5 min. Tissue sections were incubated with the blocking solution containing 10% fetal bovine serum and 1% BSA in TBS for 2 h at room temperature, and then incubated with primary antibodies at the indicated dilutions in the incubation solutions containing 1% BSA in TBS for 2 h at room temperature. Slides were then washed twice with 0.025% Triton-X 100 in TBS for 5 min and incubated with 0.3% H_2_O_2_ in TBS for 15 min for inactivation of endogenous peroxidase. After washing with TBS, tissue sections were incubated with secondary antibodies conjugated with horseradish peroxidase in the incubation solution for 1 h at room temperature. Slides were washed three times with TBS for 5 min, and then incubated with DAB solution for color development. The concentration of primary antibodies was as follows: rabbit anti-Fibrinogen gamma antibody 1/200 (GeneTex), rabbit anti-S100A9 antibody 1/200 (GeneTex), rabbit anti-Apolipoprotein E antibody 1/200 (GeneTex), rabbit anti-Vitronectin antibody 1/200 (GeneTex), and goat anti-Angiogenin antibody 1/100 (R & D Systems).

## Results

### Proteomic Profiles of Mycobacterial Granulomatous Lesions

**FIGURE 1:**
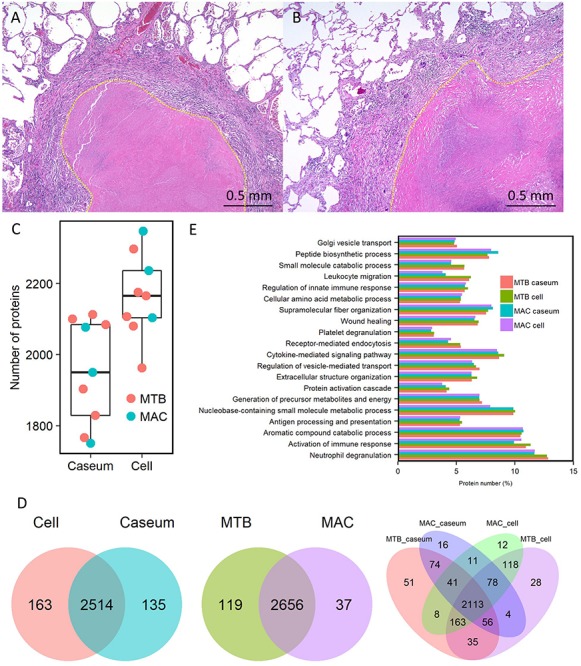
Comprehensive identification of proteins composing mycobacterial granulomatous lesions by a combination of LMD and MS-based proteomics. Representative hematoxylin-eosin (HE)-stained images of granulomatous lesions with necrotic caseum surrounded by fibrotic cells and lymphocytes caused by TB **(A)** or MAC-LD **(B)**. Granulomatous sub-compartments were separately collected by LMD followed by processing for MS-based proteomics. The boundary line between the sub-compartment of necrotic caseum and that of fibrotic cells and lymphocytes is shown (yellow dots). **(C)** Boxplot of the number of identified protein groups with LFQ values from granulomatous sub-compartments of necrotic caseum (Caseum), and fibrotic cells and lymphocytes (Cell) from TB (MTB) and MAC-LD (MAC) granulomas. Bold lines indicate the median of identified protein numbers, a box captures 50% of the measurements, and whiskers span values of the 5–95% interval. The number of proteins identified in each sample is also plotted. **(D)** Venn diagrams comparing the number of identified proteins in mycobacterial granulomatous lesions. The numbers of proteins detected in at least one sample among sub-compartments are indicated. The protein numbers identified from TB and MAC-LD granulomas (left), caseous and cellular sub-compartments of granulomas (middle) or four mycobacterial granulomatous sub-compartments (right) are indicated. MTB caseum; necrotic caseum from TB granuloma, MTB cell; cellular region from TB granuloma, MAC caseum; necrotic caseum from MAC-LD granuloma, MAC cell; cellular region from MAC-LD granuloma. **(E)** GO terms related to biological processes of identified proteins in granulomatous sub-compartments. The proportions of annotated protein numbers related to indicated GO terms are presented.

Despite their variable patterns, many lesions in human TB display a necrotic caseum (caseum) in the center surrounded by fibrotic cells and lymphocytes (cell) (Ramakrishnan, 2012; Silva Miranda et al., 2012; Lenaerts et al., 2015). As previously reported (Snijder, 1965; Marchevsky et al., 1982), the appearance of granulomatous lesions consisting of caseous and cellular sub-compartments is similar among TB and MAC-LD (Figures 1A,B). To investigate the protein composition of TB or MAC-LD granulomatous lesions, we analyzed the proteomic profiles of the caseous and cellular sub-compartments of their granulomas. We used FFPE tissues which had been surgically resected from six patients with TB and three patients with MAC-LD. The patients’ information is summarized in Table 1. Hematoxylin-stained FFPE samples were microdissected according to diagnosis by comparison with hematoxylin-eosin (HE)-stained specimens, to collect the caseous and cellular regions of granulomas separately (Supplementary Figure S1). Dissected samples were lysed with a lysis solvent, followed by trypsin digestion. Tryptic peptides were analyzed with a tandem mass spectrometer coupled to liquid chromatography (LC–MS/MS). The mass spectrometry raw data were processed using MaxQuant software against the UniProt FASTA human database. In total, 17400 peptides were identified and mapped to 2812 protein groups. The ratio of identified peptides per protein and their sequence coverage were comparable among the all samples (Supplementary Figure S2). The average numbers of identified proteins in the caseous and cellular regions of granulomas were 1952 and 2163, respectively (Figure 1C). More than 90% of the identified proteins were common to the following two groups, caseous and cellular regions, or TB and MAC-LD granulomas, and more than 75% were common to the four mycobacterial granulomatous sub-compartments (Figure 1D). GO enrichment analysis revealed that the identified proteins were mainly involved in the immune response, cellular metabolic processes, and membrane trafficking, and that their proportions were similar among four groups (Figure 1E).

**TABLE 1:** Patients’ information.

**ID**	**Disease**	**Identified bacteria**	**Age**	**Sex**	**Duration of pre-operative therapy**	**Therapy**	**Operation**	**Past history and associated information**
TB1	MDR-TB	*M. tuberculosis*	39	F	2 M	PZA/TH/CS/INH/LZD/KM	RUL	TB pleuritis
TB2	MDR-TB	*M. tuberculosis*	30	M	16 M	HREZ (4 M) REZ/LVFX (10 M) LZD/TH/EB/PAS/PZA/KM/DLM (2 M)	RUL	
TB3	DS-TB	*M. tuberculosis*	75	F	3 M	MFLX/AMK/LZD	RUL	Diabetes mellitus Drug eruption to INH/RFP/RBT/KM
TB4	MDR-TB	*M. tuberculosis*	27	F	6 M	HREZ (2 M) RBT/EB/LVFX/PZA/KM/PAS/TH (2 M) LVFX/KM/PAS/TH/CS/LZD (2 M)	RLL	
TB5	MDR-TB	*M. tuberculosis*	26	F	6 M	HREZ(2 M) KM/LVFX/TH/PAS/LZD (4 M)	Completion LLL	LUL due to trauma
TB6	MDR-TB	*M. tuberculosis*	42	M	6 M	HREZ (4 M) INH/CS/PAS/EVM/LZD (2 M)	RMLL	
MAC1	MAC-LD (FC)	MAH *M. intracellulare*	78	F	14 M	RFP/EB/CAM (12 M) RFP/EB/CAM/AMK (2 M)	RLL	
MAC2	MAC-LD (NB + FC)	MAH	67	F	96 M	RFP/EB/CAM (24 M) pause (5 M) RFP/EB/CAM (48 M) RFP/EB/CAM/STFX/SM (19 M)	LUL + S6	TB GERD
MAC3	MAC-LD (NB)	MAH	60	F	108 M	RFP/EB/CAM (12 M) pause (24 M) RFP/EB/CAM (60 M) RFP/EB/CAM/MFLX (11 M)	RML	Sinusitis

*MDR-TB, multidrug-resistant tuberculosis; DS-TB, drug-sensitive tuberculosis; MAC-LD, *Mycobacterium avium* complex lung disease; FC, fibrocavitary type; NB, nodular bronchiectatic type; MAH, *Mycobacterium avium* subsp. *hominissuis;* PZA, pyrazinamide; TH, ethionamide; CS, cycloserine; INH, isoniazid; LZD, linezolid; KM, kanamycin; RBT, rifabutin; HREZ, isoniazid + rifampcin + etanbutol + pyrazinamide; REZ, rifampcin + etanbutol + pyrazinamide; LVFX, levofloxacin; PAS, paraaminosalicylic acid; DLM, delamanid; MFLX, moxifloxacin; AMK, amikacin; EVM, enviomycin; CAM, clarithromycin; RFP, rifampicin; EB, ethanbutol; STFX, sitafloxacin; SM, streptomycin; RUL, right upper lobectomy; RLL, right lower lobectomy; LLL, left lower lobectomy; RMLL, right middle and lower lobectomy; LUL, left upper lobectomy; S6, superior segmentectomy of left lower lobe; RML, right middle lobectomy; GERD, gastro esophageal reflux disease.*

### Dynamics of Protein Composition in Granulomatous Lesions

**FIGURE 2:**
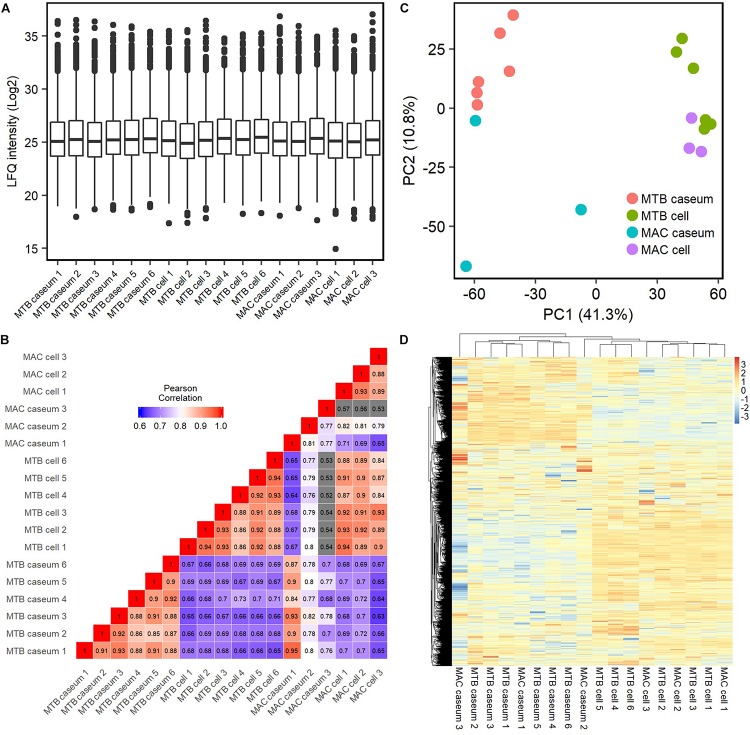
Quantitative profiling of identified proteins in granulomatous sub-compartments. **(A)** Boxplot of LFQ intensity values of identified proteins in the samples. Log_2_-transformed LFQ intensity values of identified proteins in each sample are shown. Bold lines indicate the median of log_2_-transformed LFQ intensity values, a box captures 50% of the measurements, and whiskers span values of the 5–95% interval. Outliers are shown as dots. **(B)** Heatmap of Pearson correlation coefficient (PCC) matrix of all samples. The values of PCC were generated using the log_2_-transformed LFQ values from all pairwise correlation among the samples. Coefficient numbers are also indicated. **(C)** Principal component analysis (PCA) of all samples based on LFQ intensity values. The first two main components, PC1 and PC2, accounting for 41.3 and 10.8% of data variability from all samples, are used for the plot. **(D)** Hierarchical-clustering-based heatmap for identified proteins between granulomatous sub-compartments (*n* = 2812).

To compare the protein abundance in the samples, we applied the LFQ algorithm (Cox et al., 2014). A boxplot of log_2_-transformed LFQ intensity values across the samples showed that the dynamic range of protein expression levels covered five orders of magnitude (Figure 2A). The Pearson correlation coefficient revealed that LFQ intensity values of the samples were highly correlated within the same group of the granulomatous sub-compartments, except that of the MAC-LD caseum (Figure 2B), indicating that the protein distribution is relatively homogeneous within the samples of TB caseum, TB cell and MAC-LD cell, but not within those of MAC-LD caseum. Correlation was also high between the TB and MAC-LD cellular sub-compartments. One sample of MAC-LD caseum was highly correlated to TB caseum group, whereas two other samples showed lower correlation. These results suggest that there is a heterogeneity of protein abundance in MAC-LD caseum. A PCA showed that the first component accounts for the differentiation of granulomatous lesions into caseous and cellular sub-compartments (Figure 2C). The caseous sub-compartments of TB and MAC-LD granulomas were plotted along the second component axis. In particular, the samples of MAC-LD caseum broadly distributed along the axes, reflecting low correlation among the samples of MAC-LD caseum. Hierarchical clustering analysis based on protein abundance also demonstrated that samples composed of all TB cell (*n* = 6) and MAC-LD cell (*n* = 3) and those of all TB caseum (*n* = 6) and one MAC-LD caseum (*n* = 1) formed clusters, whereas the other two samples of MAC-LD caseum did not belong to these clusters (Figure 2D and Supplementary Figure S3).

**FIGURE 3:**
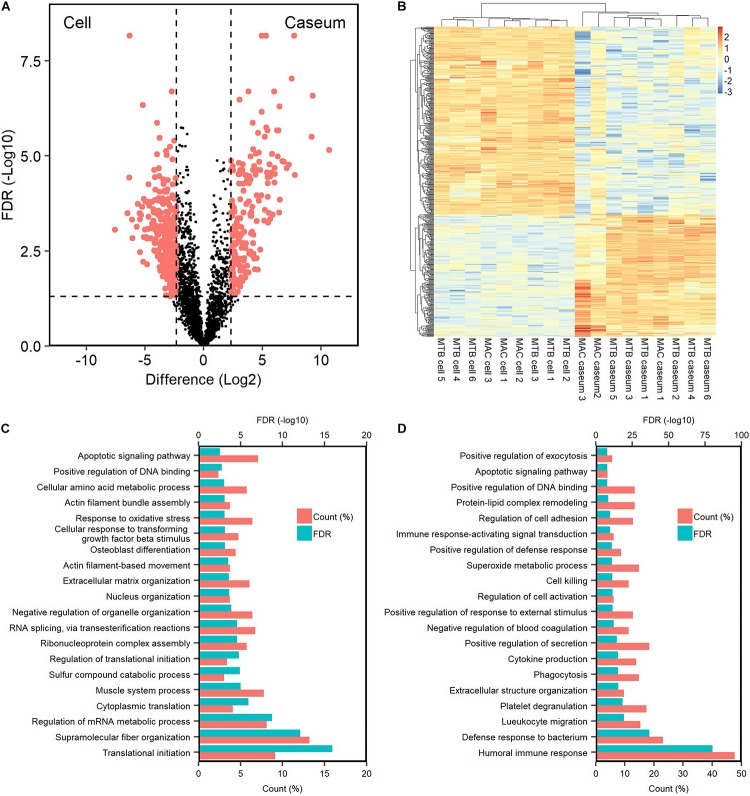
Dynamics of protein composition in granulomatous sub-compartments. **(A)** Volcano plot of protein abundance between caseous and cellular sub-compartments of granulomatous lesions combined with both TB and MAC-LD. The logarithmic ratios of average fold changes and negative logarithmic FDR values of the Welch’s *t*-test between samples from caseous and cellular sub-compartments are plotted on the *x-* and *y*-axes, respectively. Highlighting dots correspond to proteins with significantly different abundance (FDR < 0.05 and absolute value of fold change > 5). **(B)** Hierarchical-clustering-based heatmap for proteins with different abundance between granulomatous sub-compartments (*n* = 484). GO terms related to biological processes of significantly abundant proteins in Caseum **(C)** or Cell **(D)**. Top 20 ranked GO terms are listed. The proportion of identified protein numbers related to indicated GO terms and FDR are also shown.

**FIGURE 4:**
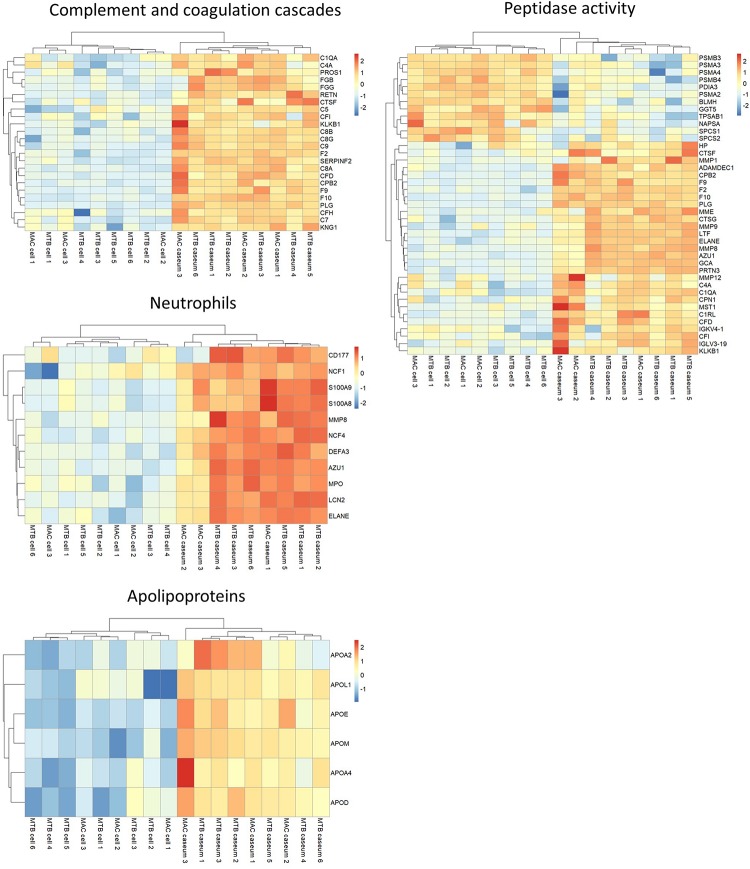
Heatmap based on LFQ intensity values of selected proteins. Hierarchical-clustering-based on LFQ intensity values of proteins involved in complement and coagulase cascades, neutrophils, apolipoproteins and peptidase activity are presented. Names of proteins involved in the pathways are also indicated.

We investigated the proteins characterizing the differentiation of granulomatous lesions into caseous and cellular sub-compartments. A volcano plot showed that 484 proteins had significantly altered abundant levels. In the caseous sub-compartments 187 proteins were significantly abundant, and 297 proteins were significantly abundant in the cellular sub-compartments (Figure 3A). Hierarchical cluster analysis clearly demonstrated the difference of protein abundance between caseous and cellular regions of TB and MAC-LD granulomas (Figure 3B). We annotated the GO terms related to biological processes for these proteins (Figures 3C,D). Proteins involved in cellular metabolic processes including transcription and translation were abundant in the cellular region. In the caseous region, proteins involved in antimicrobial responses such as defense response to bacterium, phagocytosis, and superoxide synthesis were enriched. We also found that proteins involved in complement and coagulation cascades, neutrophils and apolipoproteins accumulated in the caseum (Figure 4). Proteins annotated with peptidase activity were examined. Hierarchical clustering analysis demonstrated that the proteins also involved in proteasome (PSMB3, PSMA3, PSMA4, PSMB4) were accumulated in the cell regions, and those involved in complement and coagulation cascades, and neutrophils activity were enriched in the caseous regions. These results are consistent with the histological properties of mycobacterial granulomatous lesions (Ehlers and Schaible, 2012; Guirado and Schlesinger, 2013).

**FIGURE 5:**
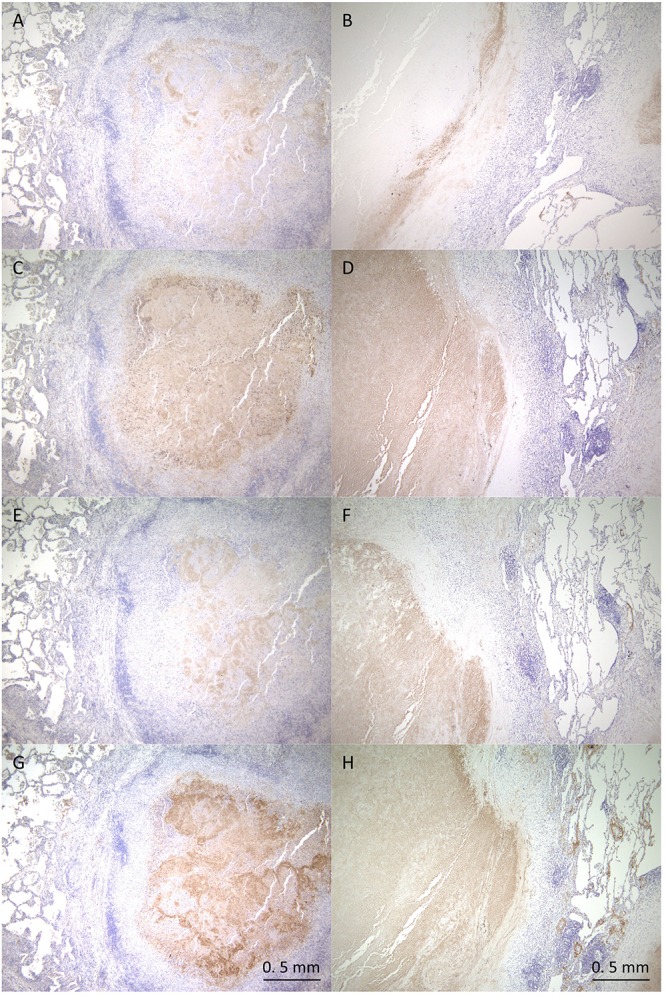
Immunohistochemistry of proteins abundant in the caseous regions. FFPE are stained with anti-Fibrinogen gamma antibody **(A,B)**, anti-S100A9 antibody **(C,D)**, anti-ApoE antibody **(E,F)** or anti-Vitronectin antibody **(G,H)**. Images of granulomatous lesions from TB **(A,C,E,G)** or MAC-LD **(B,D,F,H)** are shown.

To verify the proteomics profiling of granulomatous lesions, we examined the localization of selected proteins found to be significantly enriched in the caseous sub-compartment by immunohistochemistry (IHC) for TB and MAC-LD granulomatous lesions (Figure 5). IHC for Fibrinogen gamma (FGG), S100A9, Apolipoprotein E (ApoE), and Vitronectin (VTN) showed strong signals specifically in the caseous regions of both TB and MAC-LD granulomatous lesions, consistent with the results of proteomic profiling. These results suggest that these are representative proteins accumulated in the caseum.

### Heterogeneity in the Caseum of MAC-LD Granuloma

**FIGURE 6:**
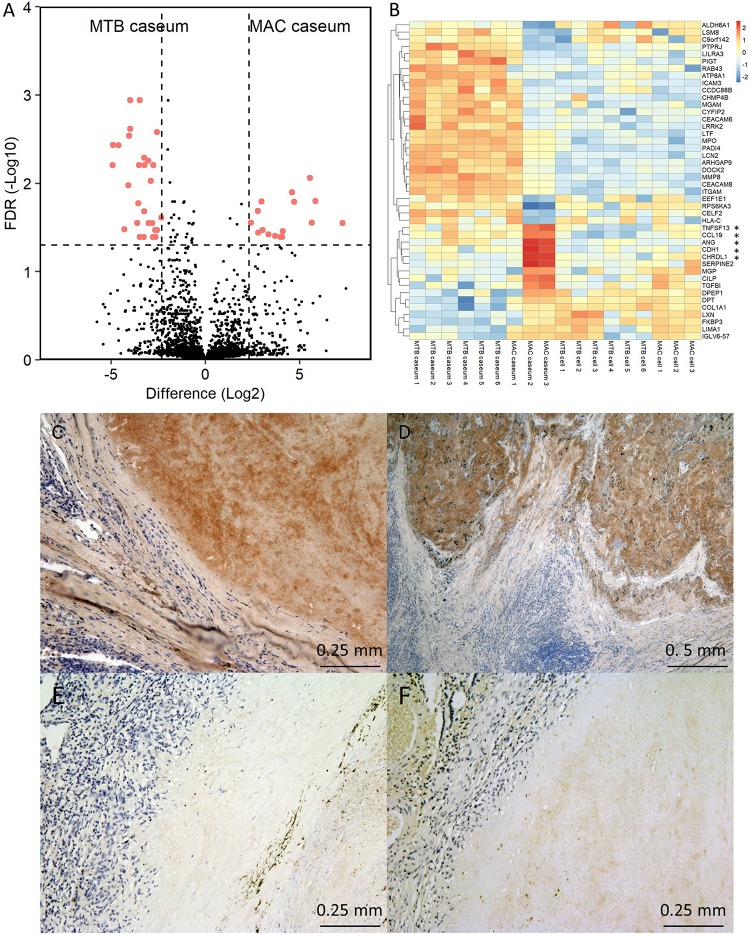
Heterogeneity of MAC-LD granulomatous lesions. **(A)** Volcano plot of protein abundance between TB caseum and selected MAC-LD caseum. The logarithmic ratios of average fold changes and negative logarithmic FDR values of the Welch’s *t*-test between samples from TB caseum and selected MAC-LD caseum are plotted on the *x-* and *y*-axes, respectively. Highlighted dots correspond to proteins with significantly different abundance (FDR < 0.05 and absolute value of fold change > 5). **(B)** Hierarchical-clustering-based heat map for proteins with significantly different abundance between TB caseum and selected MAC-LD caseum (*n* = 44). Protein names with significantly different abundance in the caseous sub-compartments from TB or MAC-LD are indicated. ^∗^Proteins with significant abundance in the selected MAC-LD caseum (*P* < 0.05, Tukey–Kramer *post hoc* test following one-way ANOVA). Immunohistochemistry of Angiogenin in granulomatous lesions of selected MAC-LD **(C,D)**, non-selected MAC-LD **(E)** and TB **(F)**.

We further examined the protein abundance in granulomatous lesions to evaluate their heterogeneity. We compared protein abundance between the caseous and cellular sub-compartments of TB granulomas (Supplementary Figure S4). The significantly abundant proteins in the caseous regions of TB were involved in the immune response and those in the cellular regions of TB were involved in cell metabolic processes. To investigate the determinants of the heterogeneity of MAC-LD granulomas, we compared protein abundance between the caseous and cellular regions of MAC-LD granulomas (Supplementary Figure S5) and between the caseous regions of TB and those of MAC-LD (Supplementary Figure S6). We found that few proteins were significantly accumulated in the MAC-LD caseum. This could be caused by the lower correlation of the protein abundance among the samples from the MAC-LD caseum (Figure 2). Therefore, we selected two samples from the MAC-LD caseum as a subgroup in which proteomic profiles were distinct from those of the TB caseum, showing relatively weak correlations in proteins (Figure 2B) and compared the protein abundance between the caseous region of TB and the selected MAC-LD. In this comparison we found that 28 proteins showed significant accumulation in the TB caseum and 16 proteins showed significant accumulation in the selected MAC-LD caseum (Figure 6A). Plotting these abundant proteins on PCA based on LFQ intensity values demonstrated that these proteins were responsible for the separation between the TB caseum and the selected MAC-LD caseum (Supplementary Figure S7). Among these proteins, we found that Angiogenin (ANG), Chordin-like protein 1 (CHRDL1), Cadherin-1 (CDH1), Tumor necrosis factor ligand superfamily member 13 (TNFSF13), and C-C motif chemokine 19 (CCL19) were significantly accumulated in the selected caseous region of the MAC-LD granulomatous lesions (Figure 6B). By IHC, we found that ANG specifically localized to the caseous region of the selected MAC-LD granulomatous lesions (Figures 6C–F). These results suggest that the differences in abundance of these proteins contribute to the heterogeneity of MAC-LD granulomatous lesions.

### Detection of Mycobacterial Proteins in Granulomatous Lesions by MS-Based Proteomics

**FIGURE 7:**
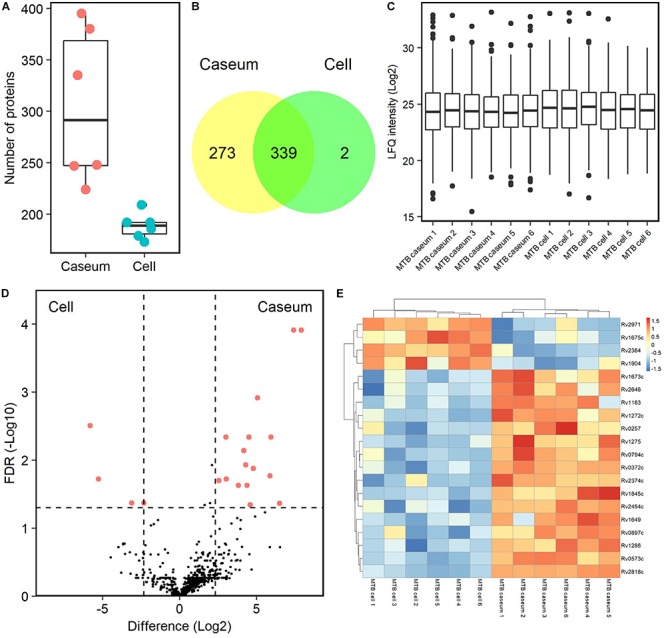
Proteomic profiling of proteins derived from *M. tuberculosis* in granulomatous lesions. **(A)** Boxplot of the number of identified *M. tuberculosis* proteins with LFQ values from MTB granulomatous sub-compartments. Bold lines indicate the median of identified number in the granulomatous sub-compartments, a box captures 50% of the measurements, and whiskers span values of the 5–95% interval. The protein number identified in each sample is also plotted. **(B)** Venn diagrams comparing the number of identified *M. tuberculosis* proteins in granulomatous lesions. The numbers of proteins detected in at least one sample among sub-compartments are indicated. **(C)** Boxplot of LFQ intensity values of identified *M. tuberculosis* proteins in samples. Bold lines indicate the median of log_2_-transformed LFQ intensity values, a box captures 50% of the measurements, and whiskers span values of the 5–95% interval. Outliers are shown as dots. **(D)** Volcano plot of protein abundance of identified *M. tuberculosis* proteins between TB granulomatous sub-compartments. The logarithmic ratios of average fold changes and negative logarithmic FDR values of the Welch’s *t*-test between samples from caseous and cellular sub-compartments are plotted on the *x-* and *y-*axes, respectively. Highlighting dots correspond to *M. tuberculosis* proteins with significantly different abundance (FDR < 0.05 and absolute value of fold change > 5). **(E)** Hierarchical-clustering-based heat map for *M. tuberculosis* with different abundance between the sub-compartments of TB granulomatous lesions (*n* = 20). Names of *M. tuberculosis* proteins with significantly different abundance are indicated.

To detect mycobacterial proteins in granulomatous lesions, the mass spectrometry raw data were processed with the MaxQuant software against the UniProt FASTA *M. tuberculosis* or *M. avium* subsp. *hominissuis* (MAH) databases. For *M. tuberculosis* proteins, 625 peptides were identified and mapped to 614 protein groups. The average numbers of identified proteins in the caseous and cellular sub-compartments of TB granulomas were about 300 and 190, respectively (Figure 7A). In caseous sub-compartments 612 proteins were identified, whereas in cellular sub-compartments 341 proteins were detected, most of which were common to both sub-compartments (Figure 7B). We next compared the expression level of identified *M. tuberculosis* proteins in the granulomatous lesions based on LFQ intensity values. The dynamic range of expression levels of *M. tuberculosis* proteins in the granulomatous lesions covered four to five orders of magnitude (Figure 7C). Twenty proteins had significantly different levels of expression, of which 16 and 4 proteins were enriched in the caseous and cellular sub-compartments of TB granulomatous lesions, respectively (Figures 7D,E and Supplementary Table S2).

**FIGURE 8:**
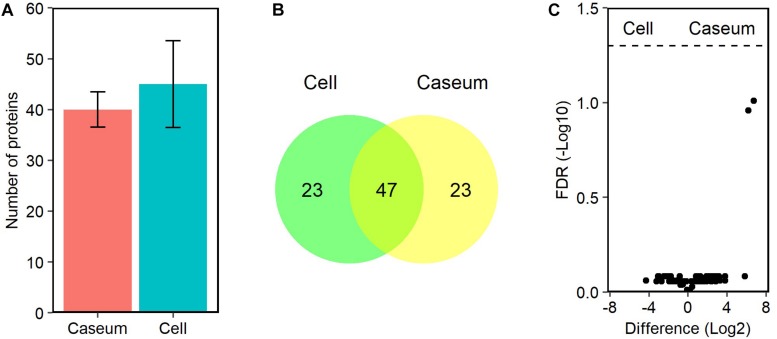
Proteomic profiles of proteins derived from MAC in granulomatous lesions. **(A)** The average number and standard deviation of identified MAC proteins with LFQ values from MAC-LD granulomatous sub-compartments. **(B)** Venn diagrams comparing the number of identified MAC proteins in granulomatous lesions. The numbers of proteins detected in at least one sample among sub-compartments are indicated. **(C)** Volcano plot of protein abundance of identified MAC proteins between granulomatous sub-compartments. The logarithmic ratios of average fold changes and negative logarithmic FDR values of the Welch’s *t*-test between samples from caseous and cellular sub-compartments are plotted on the *x-* and *y-*axes, respectively.

Because only MAH strains were isolated from patients with MAC-LD in this study (Table 1), we referred to MAH databases for detection of the MAC proteins. We identified 96 peptides mapped to 93 proteins in the MAC-LD granulomatous lesions. About 40 MAH proteins were detected among all samples of MAC-LD granulomatous lesions (Figure 8A). In both caseous and cellular sub-compartments 70 proteins were detected, of which 47 proteins were shared by each other (Figure 8B). The top 20 MAH proteins based on LFQ intensity values in the sub-compartments were identified (Supplementary Table S3). We identified no proteins with significantly different levels of the abundance in caseous or cellular sub-compartments in MAC-LD granulomatous lesions (Figure 8C).

## Discussion

To elucidate the formation process of TB granulomas, several studies have been performed by immunological or histopathological methods and microarray-based transcriptomics, in an attempt to analyze the cellular and molecular signatures of granulomas (Ulrichs and Kaufmann, 2006; Kim et al., 2010; Ehlers and Schaible, 2012; Guirado and Schlesinger, 2013; Subbian et al., 2015). A proteomic analysis of TB granulomas has also been reported (Marakalala et al., 2016). However, in the report, a batch of samples from several TB patients were mixed and analyzed, a procedure which might mask distinctive individual properties or any heterogeneity in the granulomas.

Non-tuberculous mycobacteria including MAC are opportunistic pathogens associated with pulmonary diseases in immunocompetent hosts, and disseminated infections in immunocompromised ones. The establishment of their infection in immunocompetent hosts is suggested to require one or more predisposing host conditions or comorbidities such as middle aged or elderly females, chronic obstructive pulmonary disease, cystic fibrosis, primary ciliary dyskinesia, and so on (Cook, 2010; Orme and Ordway, 2014; Swenson et al., 2018). The sites of granulomatous lesions caused by MAC-LD tend to be different from those affected by TB (Fujita et al., 1999; Hunter et al., 2007). However, the histological appearance of their granulomatous lesions has been reported to be indistinguishable (Snijder, 1965; Marchevsky et al., 1982). In this study we attempted to identify molecular signatures that distinguish TB and MAC-LD granulomatous lesions.

We identified 2812 proteins involved in granulomatous lesions in TB and MAC-LD by a combination of LMD and MS-based proteomics (Figure 1). We found the numbers of proteins identified in the caseum were significantly smaller than those in the cell (*P* = 0.004, Student’s *t*-test). This may have been caused by the partial degradation of the proteins due to the proteases released from necrotic cells in the caseum. We also identified proteins characterizing the caseous and cellular sub-compartments of granulomas by comparing their protein abundance based on LFQ intensity values (Figures 3, 4). By IHC (Figure 5), we confirmed the accumulation of FGG and VNT, which are involved in coagulation and complement cascades (Furie and Furie, 1988; Fay et al., 1999), in the caseum. Initiation of coagulation has been reported to decrease the burden of infected *M. tuberculosis* by macrophage activation in mice (Venkatasubramanian et al., 2016). These results suggest that coagulation in the caseum could be a defense mechanism against infecting mycobacteria. S100A9 proteins were specifically accumulated in the caseum of both TB and MAC-LD. This result is consistent with a previous report that S100A9 accumulated in the caseum of TB granulomas (Yoshioka et al., 2016). Proteins mainly expressed in neutrophils, including S100A9, were accumulated in the caseous sub-compartments of TB and MAC-LD granulomas, suggesting that neutrophils infiltrate into the caseum, and exacerbate pathogenesis (Dallenga and Schaible, 2016; Remot et al., 2019). We showed that ApoE and other apolipoproteins were abundant in the caseum. Apolipoproteins are regulators of cholesterol transport and metabolism (Martins et al., 2009). Considering that a hypercholesterolemic situation in ApoE knockout mice showed increased *M. tuberculosis* growth and exacerbated lung pathology (Martens et al., 2008), our results suggest that upregulation of cholesterol transport and metabolism in the caseum is involved in the regulation of mycobacterial pathogenesis. Mycobacterial granulomas are composed of a variety of cell types (Russell, 2007; Ramakrishnan, 2012). Our proteomics results also reflected the composing cell types (Figure 4). GO enrichment analysis revealed that neutrophil proteins were accumulated in the caseum region, showing that the neutrophils infiltrate into the caseum. Proteins in proteasome were abundant in the cell region, suggesting the antigen processing and presentation by dendritic cells and macrophages actively occur in this region.

To investigate the proteins involved in the heterogeneity of MAC-LD caseum (Figure 2), we compared protein abundance between selected samples of MAC-LD caseum and the TB caseum (Figure 6). In this comparison we found several proteins displaying a difference in abundance. ANG is a potent stimulator of new blood vessels through the process of angiogenesis (Tello-Montoliu et al., 2006). Angiogenesis has been reported to promote mycobacterial growth in granuloma formation (Oehlers et al., 2015), suggesting that ANG stimulates the proliferation of infecting mycobacteria in the caseum. This protein is also reported to display microbicidal activity against bacterial and fungal pathogens (Hooper et al., 2003), suggesting that ANG contributes to the inhibition of the extracellular growth of mycobacteria in the caseum. CHRDL1 is also reported to be involved in the regulation of retinal angiogenesis in response to hypoxia (Kane et al., 2008), suggesting that this protein also regulates angiogenesis within the caseum, where the hypoxic condition exists (Via et al., 2008). TNFSF13 and CCL19 have been reported to be upregulated in granulomatous lesions during their formation (Mehra et al., 2013). According to the clinical information (Table 1), NB type and duration of pre-operative therapy were characteristic in the two patients of MAC-LD whose protein abundance in the caseum displayed the heterogeneity. Overall, the proteins identified as being involved in granulomatous heterogeneity are expected to be representative markers for the pathological diagnosis in MAC-LD.

Detection of *M. tuberculosis* proteins and measurements of their expression has been reported in granulomatous lesions, especially in the caseous region (Seiler et al., 2003; Ulrichs et al., 2004; Rachman et al., 2006). Here, we demonstrated the expression of mycobacterial proteins by proteomic profiling. By comparing the LFQ intensity values of identified *M. tuberculosis* proteins, we found that several proteins were significantly abundant in the caseous and cellular regions of TB granulomas (Figure 7). Three oxidoreductases (Rv0794c, Rv0897c, Rv2971) and two transcriptional factors (Rv1675c, Rv2374c) accumulated differently in the caseous and cellular compartments. Rv2384 was mainly detected in the cellular region, which is involved in mycobactin biosynthesis (Quadri et al., 1998). Mycobactin is a siderophore, which transports free iron ions into the cytoplasm of *M. tuberculosis* cells and is involved in pathogenesis (Reddy et al., 2013). Rv1272c, a drug ABC transporter ATP-binding protein, was detected mainly in the caseum region. This protein has been also reported to function in lipid incorporation (Martin and Daniel, 2018). These results suggest that *M. tuberculosis* bacilli in caseous or cellular regions of TB granulomatous lesions adapt themselves to the environment by differential gene expression.

Mycobacteria have several protein secretion systems including type VII secretion system for their housekeeping growth and modulation of infected host immune system (Ligon et al., 2012). By comparison with secreted proteins of *M. tuberculosis* reported previously (Penn et al., 2018; Stamm et al., 2019), we found that twenty-two *M. tuberculosis* proteins among 614 detected ones were indicated to be presumably secreted proteins (Supplementary Figure S8 and Supplementary Table S4). Six of these proteins are Mce family proteins which are secreted or on the cell surface to facilitate host cell entry (Chitale et al., 2001; Saini et al., 2008; Zhang et al., 2018). These secretion systems and their substrates are considered to be potential targets for drugs and vaccines (Feltcher et al., 2010).

For detection of MAC proteins, we used protein databases of MAH strains, lineages of which have been endemic in East Asia (Uchiya et al., 2013; Yano et al., 2017) (Figure 8). Among the identified MAH proteins, acyl-[acyl-carrier-protein] thioesterase is involved in fatty acid biosynthesis. By BLAST analysis, we identified EspG and EccD of MAH as showing the greatest similarity to ESX-2 secretion-associated protein EspG2 and ESX-5 secretion system protein EccD in *M. tuberculosis*, respectively. The function of ESX-2 type VII secretion system is unknown, but, ESX-5 is involved in secretion of the mycobacteria-specific PE and PPE proteins and cell wall stability (Groschel et al., 2016). Molybdopterin molybdenumtransferase is involved in molybdenum cofactor biosynthesis. A homolog of *M. tuberculosis* has been reported as being required for survival in primary macrophages (Rengarajan et al., 2005). We identified fewer MAH proteins in MAC-LD granulomas than *M. tuberculosis* proteins in TB granulomas, in contrast with the identified proteins derived from the host (Figure 1). This could be caused by relatively paucibacillary infection of MAH within MAC-LD granulomas compared with TB granulomas.

## Conclusion

We investigated the proteomic profiles of TB and MAC-LD granulomatous lesions. Proteins involved in antimicrobial responses and cellular metabolic processes had different abundances in the caseous and cellular regions of granulomas. Among these proteins, we found representative proteins in the caseum by IHC. We also detected mycobacterial proteins in the granulomas. The results of this study provide new insights into the architecture of granulomatous lesions in TB and MAC-LD.

## Data Availability Statement

The datasets generated for this study can be found in the ProteomeXchange/jPOST; PXD014086/JPST000609.

## Ethics Statement

The studies involving human participants were reviewed and approved by Fukujuji Hospital Institutional Review Board, Research Institute of Tuberculosis Institutional Review Board. The patients/participants provided their written informed consent to participate in this study.

## Author Contributions

SS, KM, TY, MnH, AK, and NK designed and revised the project. KM, TY, MyH, FK, YS, and AK contributed clinical samples. SS and TN performed the experiments. SS, MnH, TN, and NK wrote and revised the manuscript.

## Conflict of Interest

The authors declare that the research was conducted in the absence of any commercial or financial relationships that could be construed as a potential conflict of interest.

## Supplementary Material

FIGURE S1 Separation of sub-compartments of mycobacterial granulomatous lesions by LMD. For processing of samples with the lysis solvent, FFPE were stained with hematoxylin, followed by separation of granulomatous sub-compartments by LMD. HE-stained images were prepared for the diagnosis to separately collect granulomatous sub-compartments.

FIGURE S2 Proteomics characterization of the granulomatous sub-compartments. **(A)** Number of identified peptides per protein in the samples. The average numbers in sub-compartments are described, with standard deviations. **(B)** Protein sequence coverage by identified peptides in the samples.

FIGURE S3 Dendrogram of samples by hierarchical clustering analysis based on LFQ intensity values. Cluster dendrogram of samples in Figure 2D is shown.

FIGURE S4 Protein composition in TB granulomatous lesions. **(A)** Volcano plot of protein abundance between caseous and cellular regions of TB granulomatous lesions. Highlighting dots correspond to proteins with significantly different abundance (FDR < 0.05 and absolute value of fold change > 5). GO terms related to biological processes of abundant proteins in caseous **(B)** or cellular **(C)** regions of TB granulomatous lesions. Top 20 ranked GO terms are listed. The proportion of identified protein numbers related to indicated GO terms and FDR are also shown.

FIGURE S5 Identification of proteins with a different abundance between caseous and cellular regions of MAC-LD granulomatous lesions. Highlighting dots correspond to proteins with significantly different abundance (FDR < 0.05 and absolute value of fold change > 5).

FIGURE S6 Volcano plot showing one protein with different abundances between the caseous regions of TB and those of MAC-LD.

FIGURE S7 Proteins driving separation between caseous regions of TB and those of selected MAC-LD. Components constituting PC1 and PC2 in Figure 2C are plotted. Proteins with significantly different abundance between the caseous regions are highlighted.

FIGURE S8 Venn diagram illustrating the number of proteins in common among the present (This_study) and previously reported studies (Penn et al., 2018; Stamm et al., 2019). The gene lists for Stamm_2019 and Penn_2018 were reported by Penn et al. (2018) and Stamm et al. (2019).

TABLE S1 Excel file of the raw and imputed data of all proteomic analyses for identification of human, *M. tuberculosis*, and MAH proteins in mycobacterial granulomatous lesions.

TABLE S2 Identified *M. tuberculosis* proteins in TB granulomas. *M. tuberculosis* proteins significantly abundant in caseous or cellular sub-compartments of TB granulomas are listed.

TABLE S3 Identified MAH proteins in MAC-LD granulomas. Top 20 MAH proteins with LFQ intensity values are listed.

TABLE S4 List of presumably secreted proteins of *M. tuberculosis* in Supplementary Figure S8.
