# Supplementary figures and images for: Proteomic Profiling Reveals the Architecture of Granulomatous Lesions Caused by Tuberculosis and Mycobacterium avium Complex Lung Disease
Source: Front Microbiol. 2020 Jan 17;10:3081. doi: 10.3389/fmicb.2019.03081 (PMC6978656; doi:10.3389/fmicb.2019.03081)

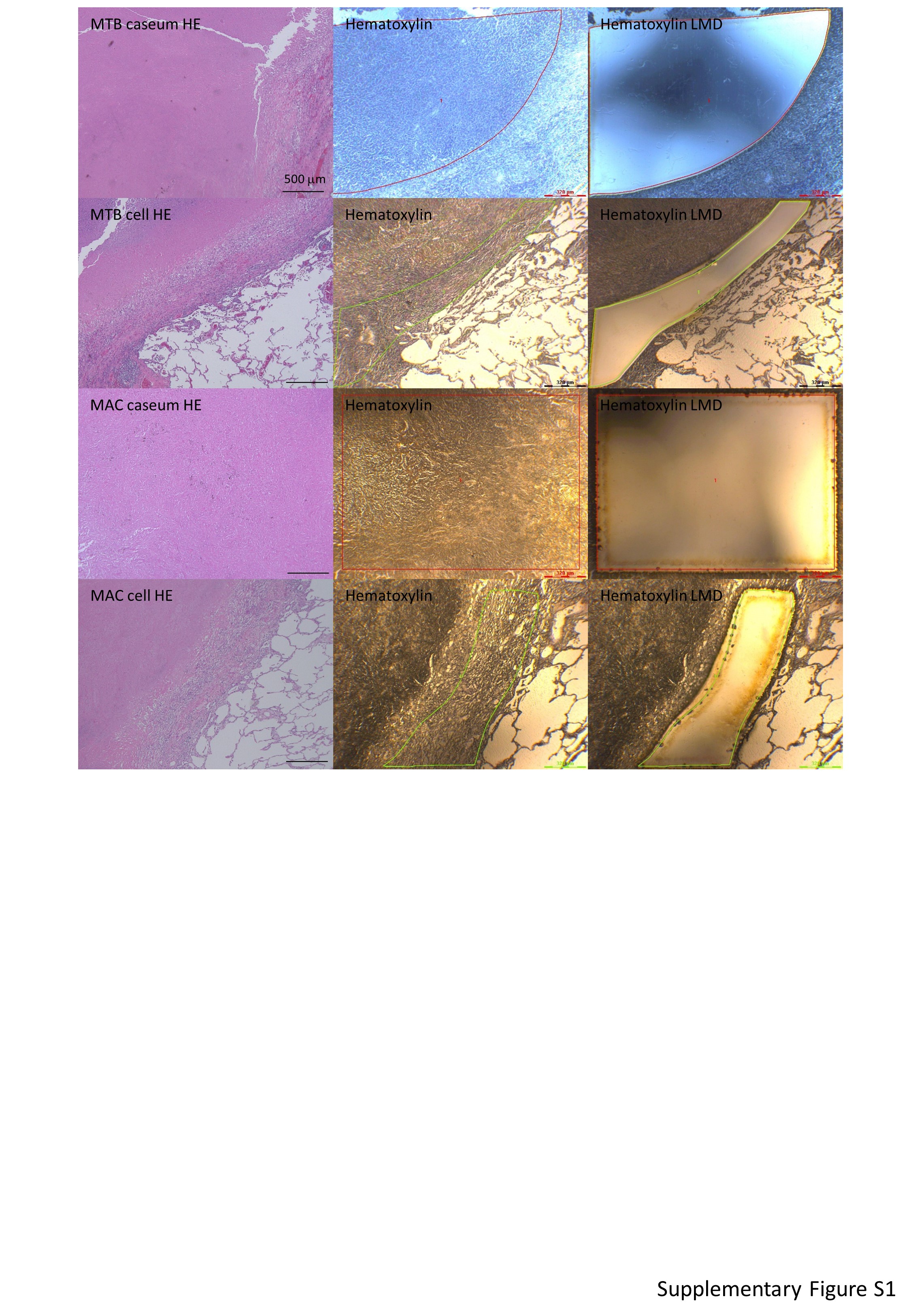

Supplement: FIGURE S1 — Separation of sub-compartments of mycobacterial granulomatous lesions by LMD. For processing of samples with the lysis solvent, FFPE were stained with hematoxylin, followed by separation of granulomatous sub-compartments by LMD. HE-stained images were prepared for the diagnosis to separately collect granulomatous sub-compartments. [file Image_1.JPEG]

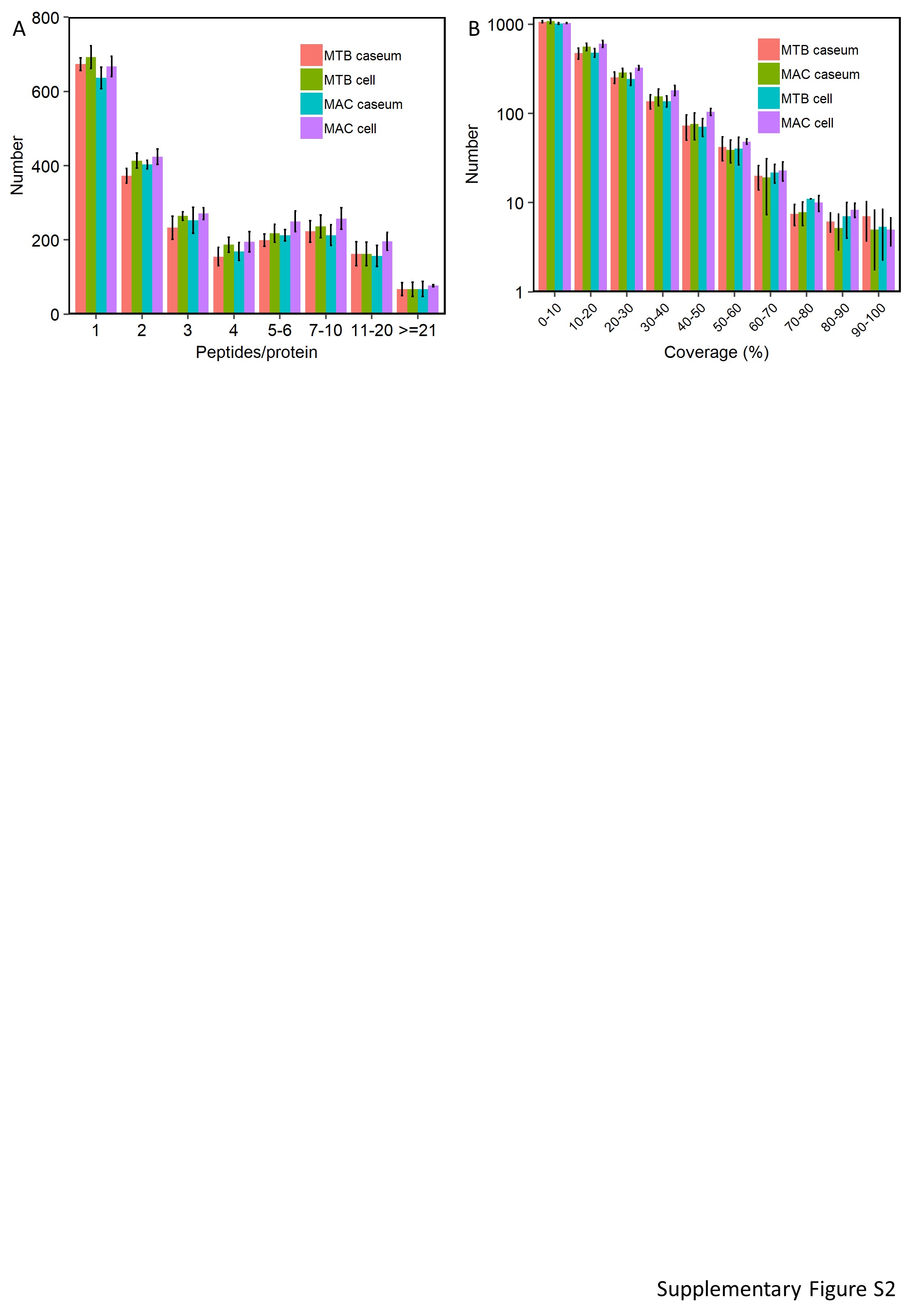

Supplement: FIGURE S2 — Proteomics characterization of the granulomatous sub-compartments. (A) Number of identified peptides per protein in the samples. The average numbers in sub-compartments are described, with standard deviations. (B) Protein sequence coverage by identified peptides in the samples. [file Image_2.JPEG]

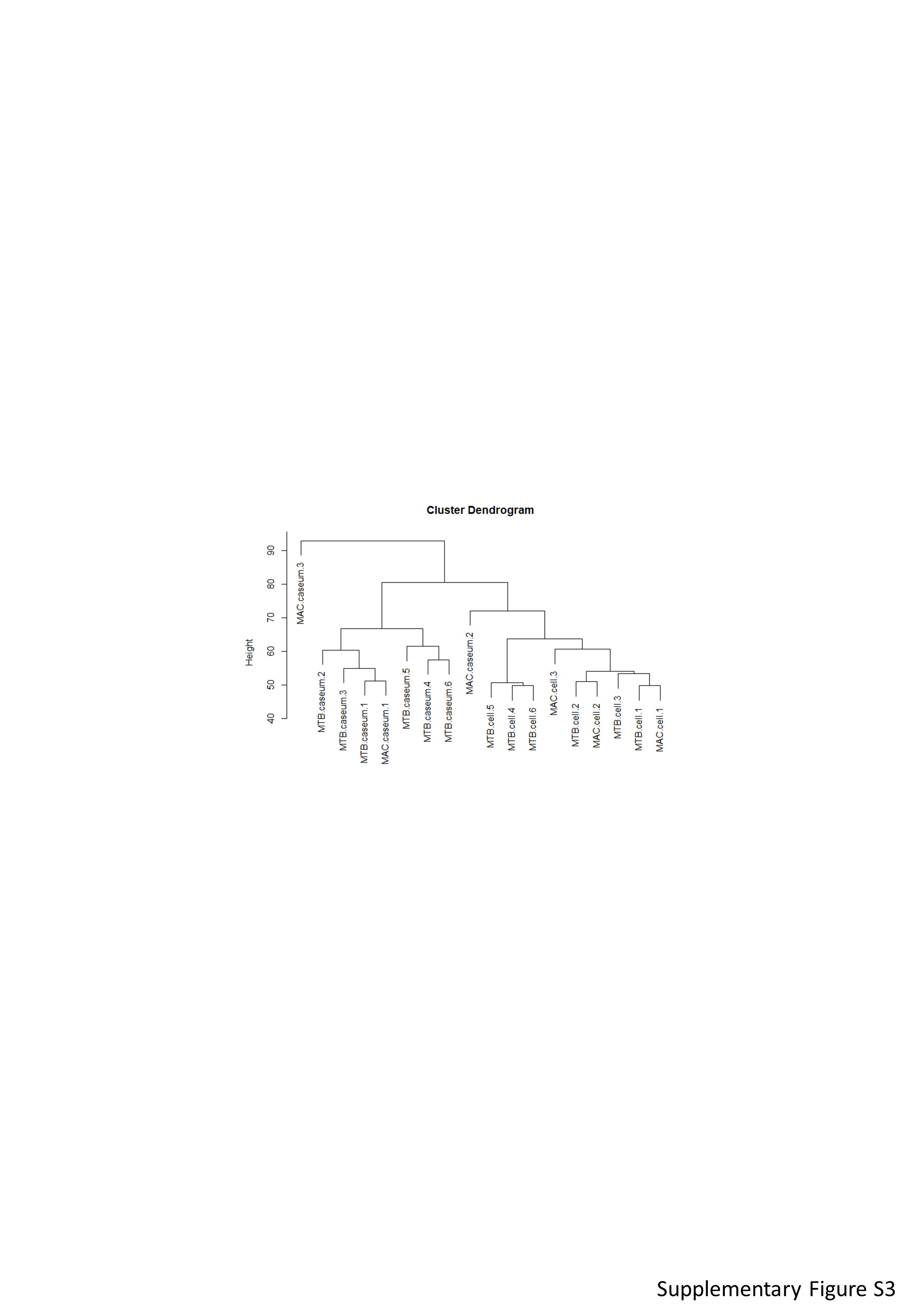

Supplement: FIGURE S3 — Dendrogram of samples by hierarchical clustering analysis based on LFQ intensity values. Cluster dendrogram of samples in Figure 2D is shown. [file Image_3.JPEG]

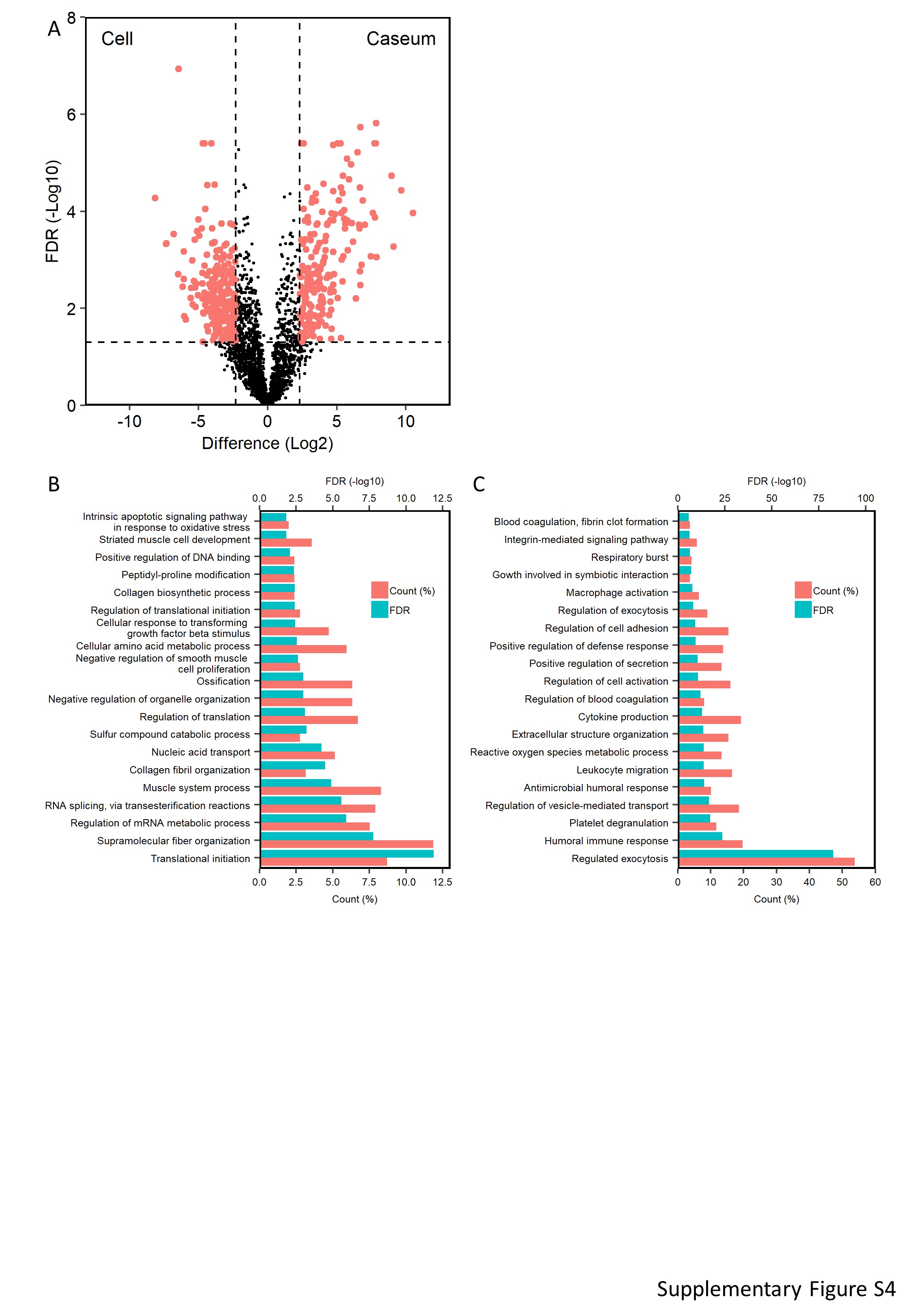

Supplement: FIGURE S4 — Protein composition in TB granulomatous lesions. (A) Volcano plot of protein abundance between caseous and cellular regions of TB granulomatous lesions. Highlighting dots correspond to proteins with significantly different abundance (FDR < 0.05 and absolute value of fold change > 5). GO terms related to biological processes of abundant proteins in caseous (B) or cellular (C) regions of TB granulomatous lesions. Top 20 ranked GO terms are listed. The proportion of identified protein numbers related to indicated GO terms and FDR are also shown. [file Image_4.JPEG]

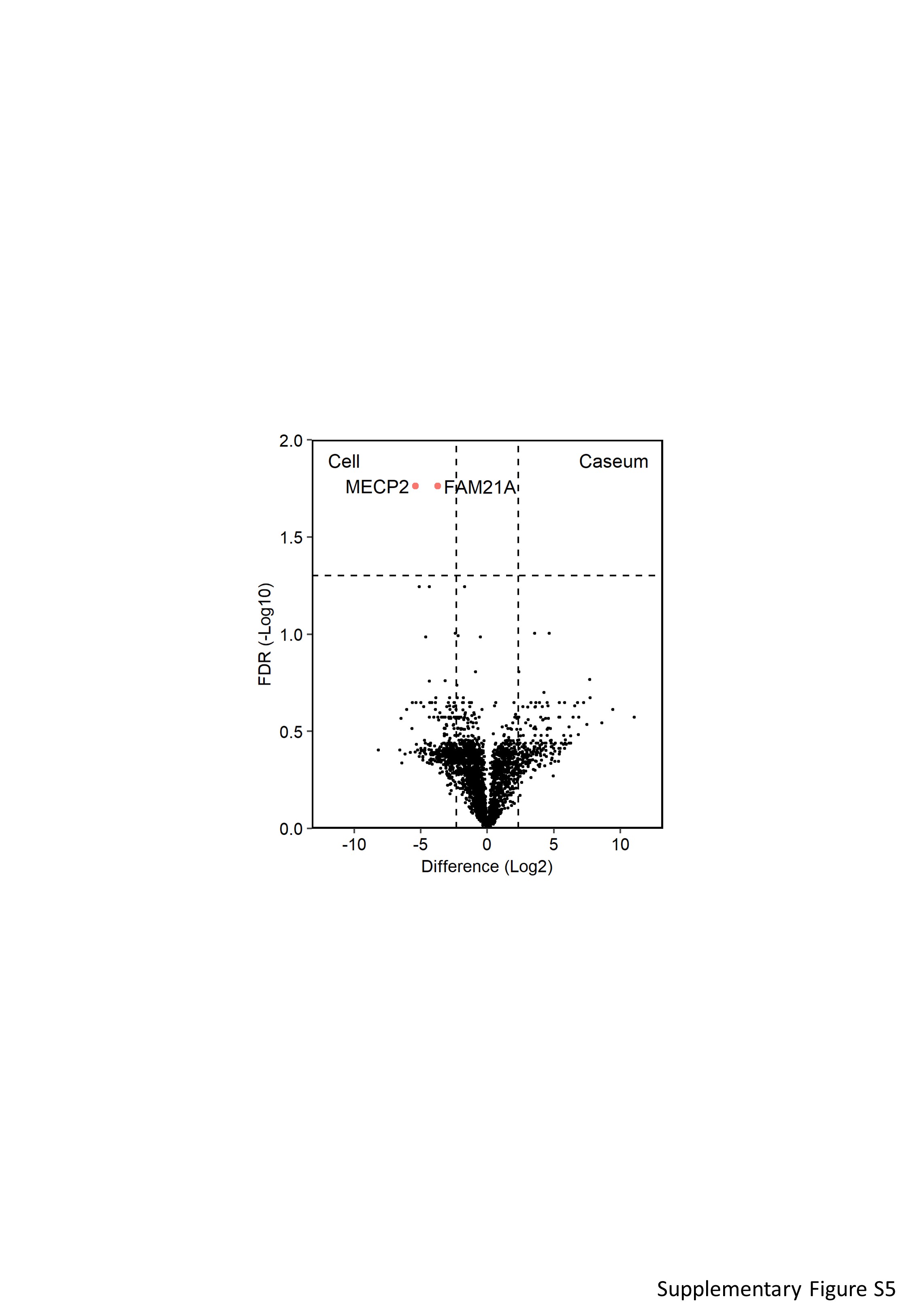

Supplement: FIGURE S5 — Identification of proteins with a different abundance between caseous and cellular regions of MAC-LD granulomatous lesions. Highlighting dots correspond to proteins with significantly different abundance (FDR < 0.05 and absolute value of fold change > 5). [file Image_5.JPEG]

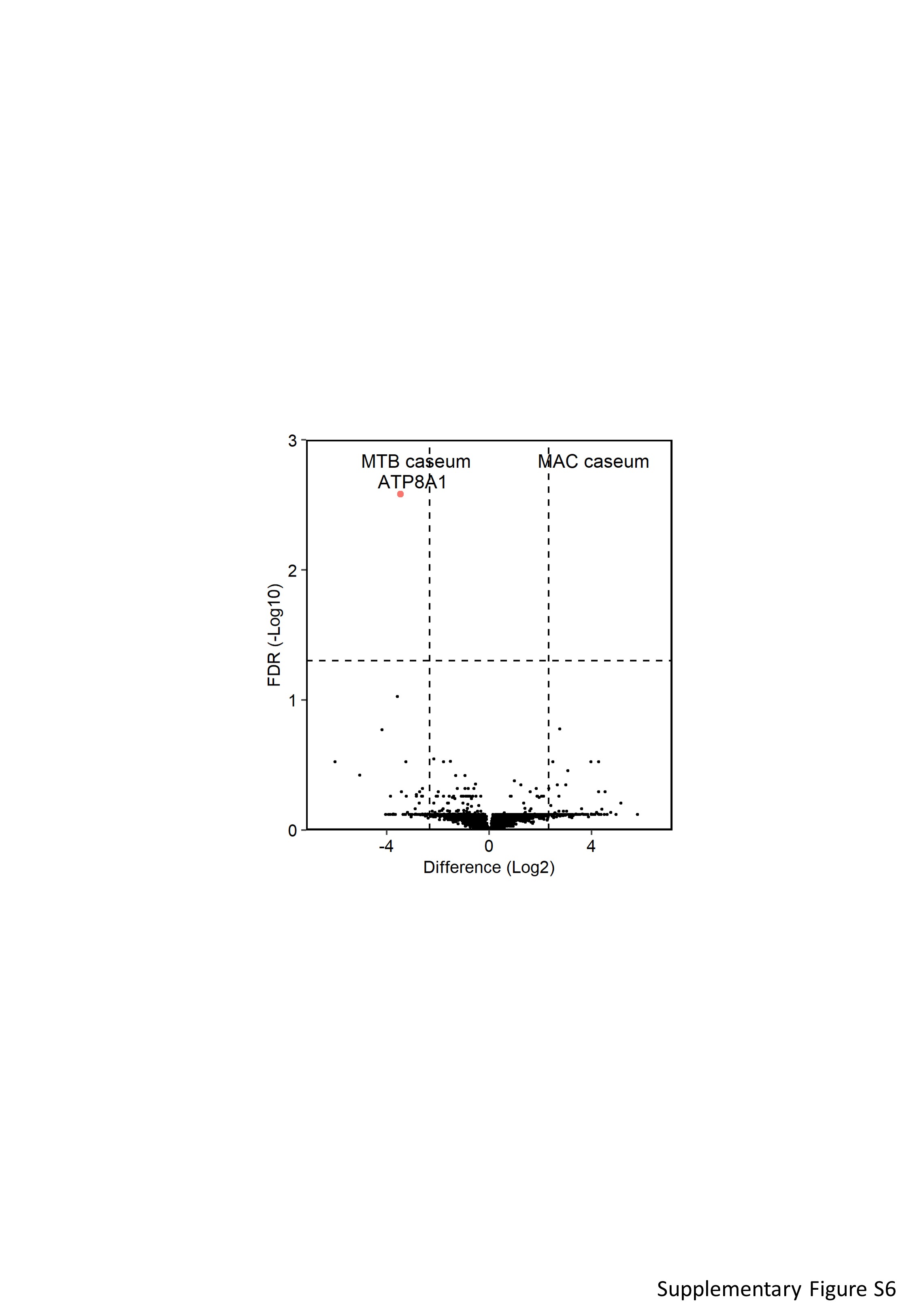

Supplement: FIGURE S6 — Volcano plot showing one protein with different abundances between the caseous regions of TB and those of MAC-LD. [file Image_6.JPEG]

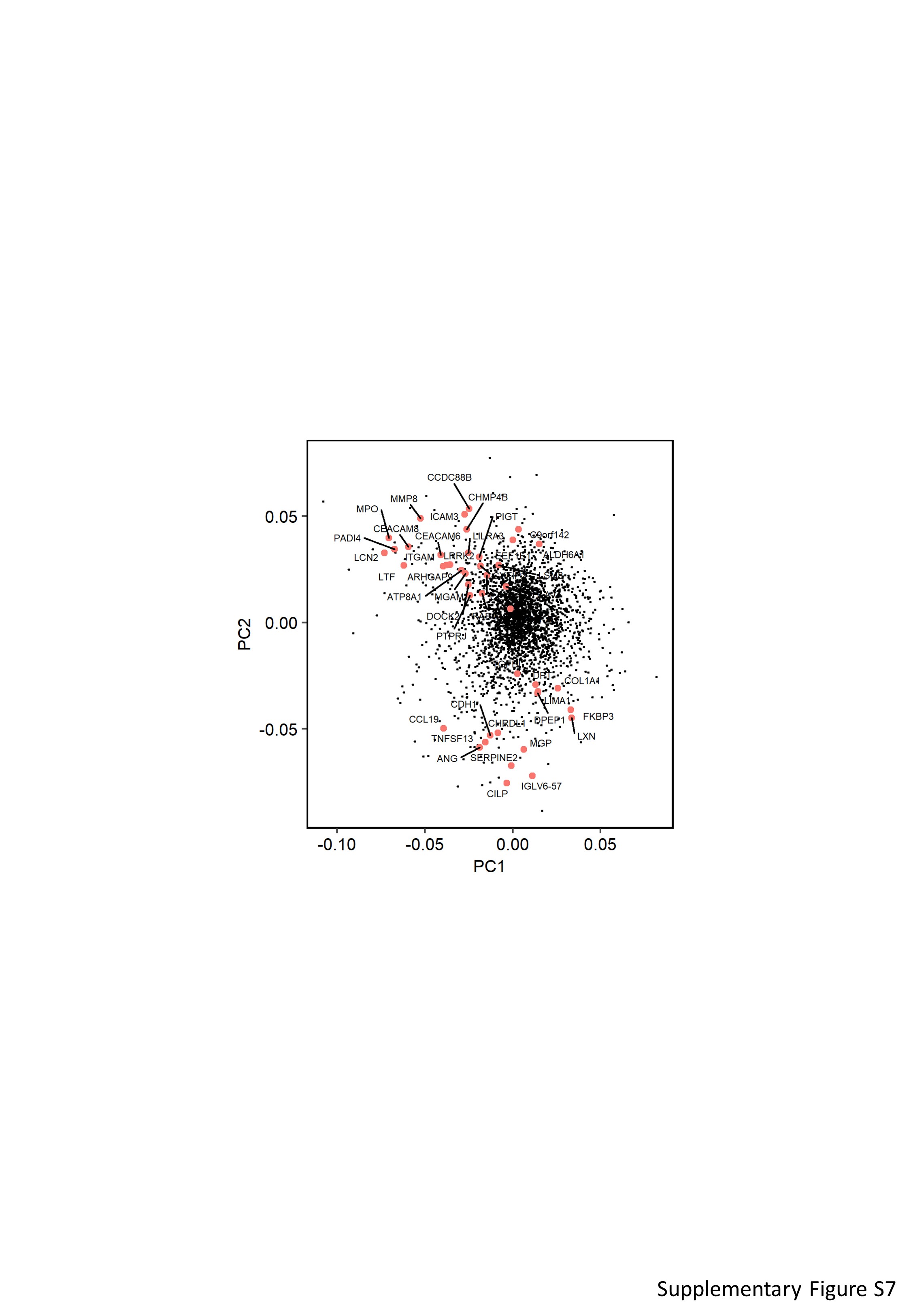

Supplement: FIGURE S7 — Proteins driving separation between caseous regions of TB and those of selected MAC-LD. Components constituting PC1 and PC2 in Figure 2C are plotted. Proteins with significantly different abundance between the caseous regions are highlighted. [file Image_7.JPEG]

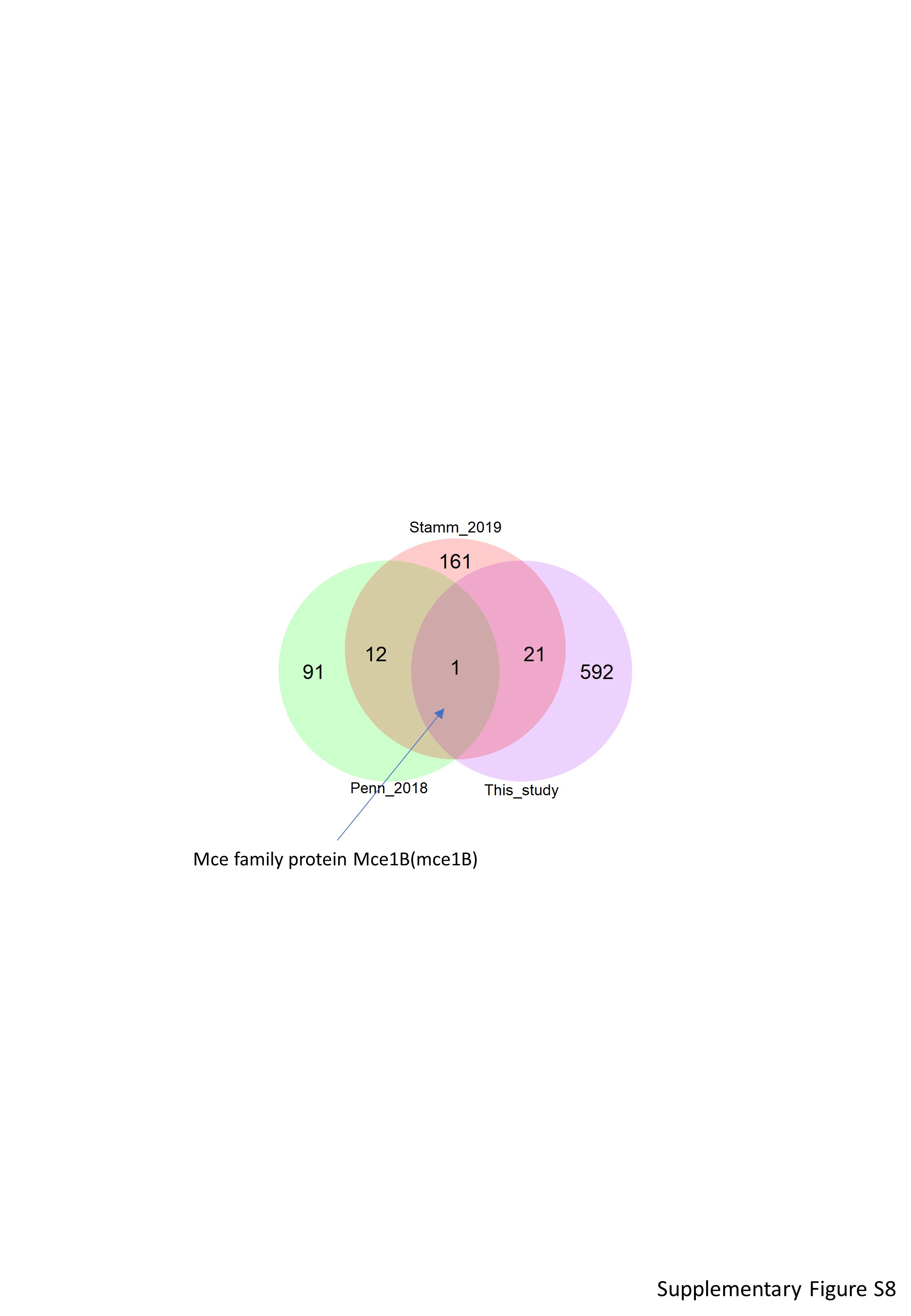

Supplement: FIGURE S8 — Venn diagram illustrating the number of proteins in common among the present (This_study) and previously reported studies (Penn et al., 2018; Stamm et al., 2019). The gene lists for Stamm_2019 and Penn_2018 were reported by Penn et al. (2018) and Stamm et al. (2019). [file Image_8.JPEG]
